# The Heptameric SmAP1 and SmAP2 Proteins of the Crenarchaeon *Sulfolobus Solfataricus* Bind to Common and Distinct RNA Targets

**DOI:** 10.3390/life5021264

**Published:** 2015-04-21

**Authors:** Birgit Märtens, Gustavo Arruda Bezerra, Mathias Josef Kreuter, Irina Grishkovskaya, Andrea Manica, Valentina Arkhipova, Kristina Djinovic-Carugo, Udo Bläsi

**Affiliations:** 1 Department of Microbiology, Immunobiology & Genetics, Max F. Perutz Laboratories, University of Vienna, Vienna Biocenter, Dr. Bohrgasse 9, 1030 Vienna, Austria; E-Mail: Birgit.maertens@univie.ac.at; 2 Department of Structural & Computational Biology, Max F. Perutz Laboratories, University of Vienna, Vienna Biocenter, Vienna Biocenter Campus 5, 1030 Vienna, Austria; E-Mails: gustavo.bezerra@univie.ac.at (G.A.B.); mathias.j.kreuter@gmail.com (M.J.K.); irina.grishkovskaya@univie.ac.at (I.G.); 3 Department of Ecogenomics and Systems Biology, University of Vienna, Althanstrasse 14, 1090 Vienna, Austria; E-Mail: manica.andrea@gmail.com; 4 Institute of Protein Research, Russian Academy of Sciences, Pushchino, 142290 Moscow Region, Russia; E-Mail: v.arkhipova@vega.protres.ru; 5 Department of Biochemistry, Faculty of Chemistry and Chemical Technology, University of Ljubljana, Aškerčeva 5, SI-1000 Ljubljana, Slovenia

**Keywords:** *Sulfolbus solfataricus*, Sm proteins, structure

## Abstract

Sm and Sm-like proteins represent an evolutionarily conserved family with key roles in RNA metabolism. Sm-based regulation is diverse and can range in scope from eukaryotic mRNA splicing to bacterial quorum sensing, with at least one step in these processes being mediated by an RNA-associated molecular assembly built on Sm proteins. Despite the availability of several 3D-structures of Sm-like archaeal proteins (SmAPs), their function has remained elusive. The aim of this study was to shed light on the function of SmAP1 and SmAP2 of the *crenarchaeon Sulfolobus solfataricus* (Sso). Using co-purification followed by RNA_Seq_ different classes of non-coding RNAs and mRNAs were identified that co-purified either with both paralogues or solely with Sso-SmAP1 or Sso-SmAP2. The large number of associated intron-containing tRNAs and tRNA/rRNA modifying RNAs may suggest a role of the two Sso-SmAPs in tRNA/rRNA processing. Moreover, the 3D structure of Sso-SmAP2 was elucidated. Like Sso-SmAP1, Sso-SmAP2 forms homoheptamers. The binding of both proteins to distinct RNA substrates is discussed in terms of surface conservation, structural differences in the RNA binding sites and differences in the electrostatic surface potential of the two Sso-SmAP proteins. Taken together, this study may hint to common and different functions of both Sso-SmAPs in Sso RNA metabolism.

## 1. Introduction

The eukaryotic hetero-heptameric Sm proteins bind to single-stranded regions of the U1, U2, U4 and U5 small nuclear RNAs (snRNAs), which are essential for pre-mRNA splicing. In addition to the prototypical Sm proteins sixteen Lsm proteins have been identified in Eukaryotes. The two Sm motifs, which consist of two stretches of conserved residues (SM1, SM2) that are separated by a long non-conserved loop, are the hallmark of Lsm proteins [1]. The binding mode of some of these hetero-heptameric ring structures with RNA substrates has been determined by x-ray crystallography [2]. Eukaryotic Sm proteins serve as ribonucleoprotein (RNP) scaffolds that impact on various RNA-mediated processes [3], including rRNA processing by small nucleolar RNPs (snoRNPs) [4], RNase P-based splicing and maturation of tRNA [5], processing of the 3' end of histone mRNA by U7 snRNP [6,7], mRNA decapping and decay [8], and chromosome end maintenance by telomerase [9].

The bacterial Sm-like protein Hfq is involved in post-transcriptional regulation. *E. coli* Hfq hexamers have dedicated RNA binding sites, preferably binding uridine-rich stretches of small regulatory RNAs (sRNAs) around the central pore on the proximal surface (L3 face) and A-rich sequences on the distal surface (L4 face) [10]. In addition, the lateral surface of Hfq contributes as well to RNA binding and duplex formation between sRNAs and their target mRNAs [11,12].The dedicated sRNA and mRNA binding surfaces on either site of the Hfq-hexamer may serve to transiently increase the local concentration of two RNA substrates. Moreover, the inherent capacity of Hfq to induce conformational changes in RNAs together with the observed structural flexibility of RNA ligands bound to Hfq could stochastically facilitate base-pairing [13].

Although the first 3D-structures of Sm assemblies were derived from archaea, the function(s) of Sm-like archaeal proteins (SmAPs) remains unknown. The SmAPs adopt a Sm-fold composed of an N-terminal α-helix and five β-strands (Figure S1). The SmAPs differ from bacterial and eukaryotic Lsm proteins. In general, SmAPs lack a long C-terminus that is characteristic for some bacterial Hfq- and eukaryotic Sm proteins [3]. However, like eukaryotic Sm proteins, they contain a RGXX consensus sequence (Figure S1; green box) within the Sm2 motif (X = any charged amino acid residue) instead of the [Y/F]KHAI consensus motif found in several bacterial proteins, with the latter being part of the RNA-binding site on the L3 surface [3]. Another distinct feature of SmAPs is the long L4 loop connecting the Sm1 and Sm2 motifs (Figure S1). An extended loop is also found in eukaryotic Sm proteins, whereas it is absent in bacterial Hfq proteins. An elongated L4 loop has been described for the U1 snRNP-subunits SmD2 and SmB [14]. The L4 loops of the U1-snRNP subunits protrude beyond the distal face and provide one interaction site to clamp a stem-loop of the snRNA [14].

Archaea often possess two SmAP paralogues, SmAP1 and SmAP2, both of which form homo-heptamers, and occasionally rings of different sizes [3]. *Crenarchaeota* have an additional SmAP, SmAP3, which contains a conserved Sm domain linked to a second domain by a flexible linker as shown for *Pyrobaculum aerophilum* (*Pa*) SmAP3 [15,16]. *Sulfolobus solfataricus* encodes the three SmAPs, Sso 6454 (SmAP1), Sso 5410 (SmAP2) and Sso 0276 (SmAP3) (http://www-archbac.u-psud.fr/projects/sulfolobus/). Whereas SmAP1 (Sso 6454) and SmAP2 (Sso 5410) show 50% similarity and 36.4% identity (Figure S1A), their similarity and identity to Sso 0276 (SmAP3) is only 30% and 10%, respectively. The Sso-SmAP1 gene is co-transcribed with ribosomal protein gene L37e (Sso 6543) (Figure S1B). The highly conserved gene synteny is found in over 40 archaeal genomes, which might suggest a conserved role in processing or stabilization of rRNAs [17]. The Sso-SmAP1 operon, which additionally encodes a spermidine synthetase (Sso 0757), is flanked by 2 tRNAs, t33 and t34, the latter of which contains an intron (http://gtrnadb.ucsc.edu/Sulf_solf/Sulf_solf-by-locus-txt.html). The monocistronic Sso-SmAP2 gene is transcribed in the same direction as a methionine adenosyl transferase gene (MAT) (Figure S1B). Again, this genomic organization is conserved in most *crenarchaea* and *thaumarchaea* [3]. The structure of the homo-heptameric Sso-SmAP1 has been solved [18]. Several studies revealed that SmAPs from different archaea bind to oligo-U stretches of various lengths [3,17,19,20,21]. Furthermore, it has been shown by co-immunoprecipitation that the two SmAPs of *Archaeglobus fulgidus* (Af-SmAP1 and Af-SmAP2) associate with RNase P RNA *in vivo* [20], which might suggest a role in tRNA processing. Moreover, the *euryarchaeum Haloferax volcanii* (Hv) harbors only one SmAP1 protein, which was shown to associate with several uncharacterized ncRNAs, tRNAs and C/D box snoRNAs [17].

In this study, we started to characterize the function of SmAPs in the *crenarchaeum* Sso. The crystal structure of Sso-SmAP2 was determined at 2.6 Å resolution. Sso-SmAP2 forms a homo-heptameric assembly like Sso-SmAP1 [18]. However, when compared with Sso-SmAP1, structural differences were noted in the RNA binding site(s) and in the electrostatic surface potential. Moreover, RNA substrates were identified for both Sso proteins, which could provide hints for their function(s) in RNA metabolism.

## 2. Materials and Methods

### 2.1. Construction of Plasmids pMJ05-SmAP1-His and pMJO5-SmAP2-His and Transformation of Sso Cells

The Sso-SmAP1 gene (Sso 6454) and the Sso-SmAP2 gene (Sso 5410) were amplified by PCR using genomic DNA of Sso strain P2 as template. For amplification of the Sso-SmAP1 gene the oligonucleotides 5'-CATGCCATGGATTTTTTGGCAGAAACAGCGC-3' (contains a NcoI restriction site) and 5'-CGCGGATCCAGAAGTTTGTAGAGGAGAAATT-3' (contains a BamHI restriction site) were used. For the Sso-SmAP2 gene the oligonucleotides 5'-CATGCCATGGAAGCAAAAGTAGAAAATCCG-3' (contains a NcoI restriction site) 5'-CGCGGATCCTTTCTCACTATTCATAACTG-3' (contains a BamHI restriction site) were used.The PCR products were cleaved with NcoI and BamHI, and ligated into the corresponding sites of plasmid pSVA5 [22], resulting in plasmid pSVA5-SmAP1-His and pSAV5-SmAP2-His. Plasmids pSVA5-SmAP1-His and pSAV5-SmAP2-His were then cleaved with EagI and AvrII, and the resulting fragment was ligated into the corresponding sites of plasmid pMJ05 [22], which gave rise to plasmids pMJ05-SmAP1-His and pMJ05-SmAP2-His. The coding sequence for the Sso-SmAP1 and the Sso-SmAP2 genes is preceded by an inducible arabinose promotor [22].

Three hundred nanograms of the respective plasmid DNA was used for electroporation of Sso PH1-16 (∆*pyrEF*; ∆*lacS*) cells as described [22]. The cells were regenerated in medium containing uracil and then selected in medium without uracil. After reaching an OD_600_ of 0.5, the cells were plated and single colonies were inoculated in liquid media without uracil. Genomic DNA was isolated and the presence of the intact plasmid was confirmed using the pMJ05 specific primers 5'-GGATGCTAAACAACTATTCAAACTG-3' and 5'-GTTGTGTGGAATTG-TGAGCGGATAA-3'.

### 2.2. Synthesis of His-tagged Sso-SmAP Proteins and Purification

The Sso strains PH1-16 [pMJ05-SmAP1-His] and PH1-16 [pMJ05-SmAP2-His] were grown in Brock’s medium (composed of Brock’s salts and supplemented with 0.2% NZamine and 0.2% sucrose) until the cells reached an OD_600_ of 0.8. The cells were pelleted, washed twice with water and transferred to 400 mL Brock’s medium supplemented with 0.2% Nzamine and 0.2% D-arabinose (OD_600_ = 0.2). When the cells reached an OD_600_ of 0.8, the cells were harvested and lysed by sonication in 20 mL lysis buffer (50 mM Tris-HCl, pH 8.0; 0.3 M NaCl; 15 mM imidazole; 10 mM β-mercaptoethanol; 1mM PMSF). The lysate was centrifuged at 25,000 g and the supernatant was incubated with 200 µL NiNTA magnetic agarose beads (Qiagen, Hilden, Germany) overnight at 4 °C. Then, the beads were pelleted using a magnetic device. To remove non-specifically bound or weakly associated proteins or RNA, the beads were washed three times with 1 mL buffer (50 mM Tris-HCl, pH 8.0; 1 M NaCl; 40 mM imidazole in DEPC-water). RNA and protein(s) were eluted with 500 µL elution buffer (50 mM Tris-HCl, pH 8.0; 300 mM NaCl; 250 mM imidazole in DEPC-water). Fifteen microliters of the eluates were separated by SDS-PAGE (Figure 4A), whereas the rest of the eluates was extracted twice with phenol/chloroform and precipitated. The precipitated RNA was resuspended in DEPC-water.

### 2.3. Deep Sequencing Analysis

The RNA co-purifying with and eluted from either Sso-SmAPs was fragmented to an average length of 200–300 nt by incubation for 2 min at 94 °C in 40 mM Tris-acetate pH 8.2, 100 mM potassium-acetate and 30 mM magnesium-acetate [23]. The samples were cooled on ice and purified on a Sephadex G50 column. cDNA synthesis was carried out using the Invitrogen cDNA synthesis Kit. 400 ng of RNA was mixed with 1µL random hexamer primers (100 pmol/µL, Promega, Madison, WI, USA) and DEPC-H_2_O, in a total volume of 10 µL. The mixture was heated to 70 °C for 10 min and incubated on ice. Four microliters of first strand reaction buffer (Invitrogen cDNA synthesis Kit, Invitrogen, Waltham, MA, USA), 2 µL of 0.1 M DTT and 1 µL of 10 mM dNTP were added and incubated at 45 °C for 2 min. Then, 1 µL SuperScriptII RT was added and the mixture was incubated at 45 °C for 1h, and then placed on ice. Then, the following reagents were added: 91 µL DEPC-water, 30 µL second strand reaction buffer (Invitrogen cDNA synthesis Kit, Invitrogen, Waltham, MA, USA), 3 µL 10 mM dNTP, 1 µL *E. coli* DNA ligase (10 U/µL), 4 µL *E.coli* DNA polymerase I (10 U/µL) and 4 µL *E. coli* RNase H (2 U/µL), to a final volume of 250 µL. This mixture was incubated for 2 h at 16 °C, followed by addition of 2 µL of T4 DNA polymerase (10 U/µL) and incubation at 16 °C for 5 min. Then, the sample was incubated on ice and 10 µL of 0.5 M EDTA was added. The cDNA was purified using Phenol/Chloroform. Following cDNA synthesis the different samples were subjected to next generation RNA sequencing (NGS; Illumina platform GAIIx, Illumina, San Diego, CA, USA).

### 2.4. Bioinformatic Analyses

The raw reads were aligned to the SSO P2 reference genome (http://www.ncbi.nlm.nih.gov/nuccore/15896971) using the bowtie aligner (http://bowtie-bio.sourceforge.net/index.shtml). Counting of the reads, mapping to the coding regions and non-coding regions were done using the bedtools software package (http://code.google.com/p/bedtools/). Normalization and visualization was done using the R/Bioconductor software. The reads from the alignment were counted for the coding regions and for non-coding regions and then normalized to obtain the FPKM (Fragment Per Kilobase of exon model per Million mapped reads) values. The FPKM measure of read density reflects the molar concentration of a transcript in the starting sample by normalizing for RNA length and for the total read number in the measurement. This facilitates transparent comparison of transcript levels both within and between samples [23,24]. A threshold of a two-fold and eight-fold difference in the FPKM values was used to select non-coding RNAs and mRNAs, respectively that bound to either Sso-SmAP1 or to Sso-SmAP2.

### 2.5. Expression of Sso ORF 5410 in E. coli and Purification of Sso-SmAP2

Sso ORF *5410* was amplified by PCR using genomic DNA of strain Sso P2 and the oligonucleotides 5'-CCATGCCATGGATATGCAAGCAAAAGTAGAAAATCCG-3' (contains a NcoI restriction site) and 5'-GGACTAGTTTATTTCTCACTATTCATAAC-3' (contains a SpeI restriction site). The PCR product was then cleaved with the NcoI and SpeI and ligated into the corresponding sites of plasmid pPROEX-Htb (Invitrogen, Waltham, MA, USA), resulting in plasmid pPROEX-SmAP2_His_. The plasmid was transformed into *E. coli* strain JW4130∆*hfq*. Then, 8 l LB medium containing 25 µg/mL kanamycin and 100 µg/mL ampicillin at 37 °C were inoculated. The culture was cooled to 20 °C and expression of ORF *5410* was induced by adding 0.5 mM IPTG. After growth for 10 h at 20 °C, the cells were harvested and the pellet was resuspended in lysis buffer (50 mM Tris-HCl, pH 8.0; 300 mM NaCl; 15 mM imidazole; 1mM PMSF; 10 mM β-mercaptoethanol; RNaseA 10µg/mL; DNase I 10 µg/mL; 0.1% Triton X-100; 25 µg/mL lysozyme) and incubated for 20 min on ice. The cells were lysed by sonication. The lysate was centrifuged at 25.000 g for 30 min at 4 °C. The supernatant was incubated with Ni-NTA Agarose (Qiagen; 1mL per pellet derived from a 2 L culture) overnight at 4 °C. The lysate with beads was transferred to Poly-Prep chromatography columns (Bio-Rad, Hercules, CA, USA) and washed with 30 mL buffer (50 mM Tris-HCl, pH 8.0; 1 M NaCl; 40 mM imidazole; 1 mM PMSF; 10 mM β-mercaptoethanol; 0.1% Triton X-100). The bound protein was eluted in 10 mL elution buffer (50 mM Tris-HCl, pH 8.0; 300 mM NaCl; 200 mM imidazole; 10 mM β-mercaptoethanol; 0.1% Triton X-100). The eluates were dialyzed overnight at 4 °C in dialysis buffer (50 mM Tris-HCl, pH 8.0; 300 mM NaCl; 1 mM DTT; 3.4 mg TEV protease for 10 mL eluate) followed by a further incubation at 25 °C for 3 h. The eluates were concentrated to a volume of 5 mL and incubated for 20 min at 65 °C to denature the TEV protease. Then 200 µL NiNTA Agarose was added to remove uncleaved protein. The flowthrough was collected and passed through a 0.45 µm filter before the sample was further purified by size exclusion chromatography using Superdex75 16/60 column equilibrated with SEC-buffer (50 mM Tris 8.0; 200 mM NaCl; 10 mM β-mercaptoethanol). The purity of Sso-SmAP2 was assessed by SDS-PAGE and the protein preparation was resuspended to a final concentration of 10 mg/mL.

### 2.6. Crystallization, Data Collection and Structure Determination

Crystallization trials were performed using the vapor-diffusion method in a 96-well MRC-2 plate at 293 K by mixing equal volumes (0.2 µL) of 10 mg/mL protein solution and reservoir solution using a Phoenix robot (Art Robbins Instrument, Sunnyvale, CA, USA). Crystallization trials were initially set up using several commercial screens. Plate-like crystals were identified in PEGRx screen using condition 28, which contains 0.1 M citric acid (pH 3.5) and 25% w/v polyethylene glycol 3350. To optimize the crystallization conditions, trials using the hanging-drop vapor-diffusion method in Linbro plates were performed. Equal volumes (1 µL) of 10 mg/mL protein solution and reservoir solution were mixed and equilibrated against 300 µL of the latter. Crystals were flash-cooled in liquid nitrogen after being cryoprotected by passage through a solution of 0.1 M citric acid (pH 3.5), 25% w/v polyethylene glycol 3350 and 20% glycerol. Diffraction data were collected on beamline Id23-1 at the European Synchrotron Radiation Facility (ESRF). The data were processed using XDS [25] and scaled in space group H3:R with Scala [26], part of CCP4 program suite [27]. Data quality was accessed with phenix.xtriage and Pointless [28], which identified the twin law h,-h-k,-l.The structure was solved by molecular replacement using Phaser [29]; the coordinates of SmAP1 from *Methanobacterium thermautotrophicum*(PDB code: 1JRI) were used as a search template, which has 41% identity. The graphics program COOT [30] was used for manual model refinement and visualization. The model was refined with PHENIX [31], adopting TLS refinement and applying the twin law: h,-h-k,-l. R_free_ values [32] were computed from 5% randomly chosen reflections not used for refinement. The model was refined at 2.6 Å resolution, to a R-factor of 14.0% and to R_free_ of 17.7%. MolProbity [33] was used for stereochemistry analysis and structure validation. Detailed statistics of the data-collection and structure refinement are listed in Table 1. Poisson-Boltzmann calculations were performed using the software APBS [34,35] to analyze the charge distribution over the heptameric ring of Sso-SmAP1 and Sso-SmAP2. Figures were prepared using Chimera [36] and the program PyMOL (http://www.pymol.org/).

Probability measures P_ΔG,IF_ of specific interfaces were derived from the gain in solvation energy upon complex formation, with P_ΔG,IF_ > 0.5 pointing to hydrophilic/unspecific and P_ΔG,IF_ < 0.5 to hydrophobic/specific interfaces using PISA [37].

**Table 1:** Data collection and refinement statistics.

	Sso-SmAP2
Data collection	
Beamline	ESRF ID23-1
Wavelength (Å)	0.9762
Unit cell	*a* = 197.21 Å, *b* = 197.21 Å, *c* = 38.88 Å, α = 90.0°, β = 90.0°, γ = 120.0°
Space group	*R3: H*
Resolution range (Å) *	49.3-2.6 (2.74-2.6)
Completeness (%)	100 (100)
Multiplicity	5.3 (5.1)
R_sym_	0.127 (0.990)
R_pim_	0.061 (0.49)
Mean I/σ_I_	29.2 (2.1)
Unique reflections	17319 (2519)
**Refinement**	
R/R_free_ Twin_fraction	0.140/0.177 0.36
r.m.s.-deviations	
bond length (Å)	0.006
bond angle (°)	1.09
Number of atoms	
protein	44384
water	550
B-factors (Å^2^)	
protein	55.10
water	54.81

* Values for the highest resolution shell are given in parentheses.

Coordinates and structure factors have been deposited with the Protein Data Bank under the accession code 4XQ3.

## 3. Results and Discussion

### 3.1. 3D-Structure and Surface Charge of Sso-SmAP2 in Comparison with Other SmAPs

**Figure 1:**
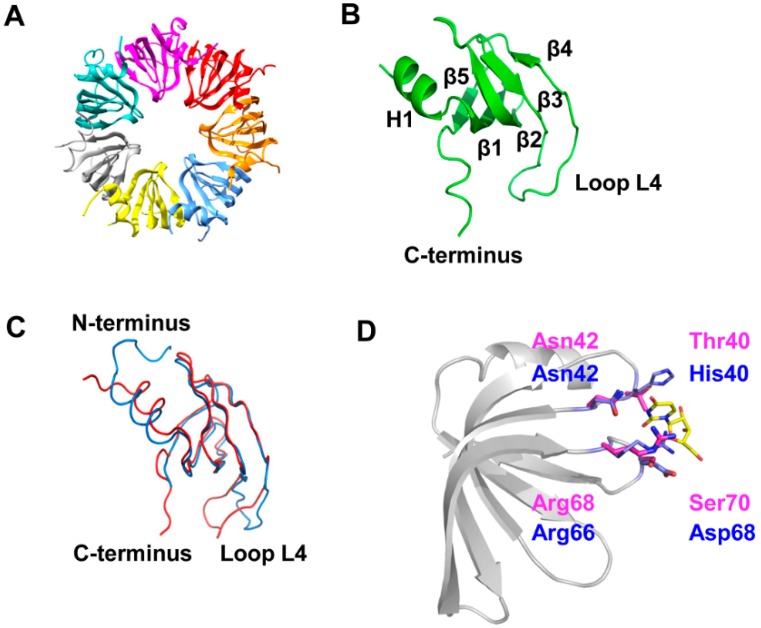
Crystal structure of Sso-SmAP2 and comparison with Sso-SmAP1. (**A**) Ribbon diagram of the heptameric Sso-SmAP2 ring. Each subunit is displayed in a different color. (**B**) Ribbon diagram of a protomer of Sso-SmAP2. Sso-SmAP2 possesses the classical Sm-fold, consisting of an N-terminal helix (H1) and five strongly bent ß-strands forming a half-open β-barrel (β 1–5). (**C**) Superposition of a single subunit of Sso-SmAP1 (blue) and Sso-SmAP2 (red). (**D**) Superposition of the putative uridine-binding pockets of Sso-SmAP1 and Sso-SmAP2. The conserved residues forming the uridine-binding pocket as described for Af-SmAP1 [18,19,20] are depicted in magenta for Sso-SmAP1 and in blue for Sso-SmAP2.

Sso-SmAP2 rhombohedral crystals (space group *R*3: H) were grown with one homo-heptameric ring in the asymmetric unit as depicted in Figure 1A. Sso-SmAP2 possesses the classical Sm fold, consisting of an N-terminal helix and five strongly bent strands forming a half-open β-barrel (Figure 1B). The heptameric assembly is formed via the outer β-strands, β4 and β5 (Figure 1B) [2], resulting in a ~12 Å central pore (Figure 1A).The overall architecture of the heptameric Sso-SmAP1 and the heptameric Sso-SmAP2 complex is very similar, as the superposition of the two heptameric assemblies yielded a root-mean-square deviation (rmsd) of 1.1 Å for 391 out of 515 superimposed Cα atoms. The superposition of the Sso-SmAP1 and Sso-SmAP2 protomers (Figure 1C) yielded a lower rmsd of 0.6 Å (over 53 out of 64 residues). The major structural differences between both Sso-SmAPs are found in loop L4 and in the N- and C-terminal regions (Figure 1C).The extended N-terminus of Sso-SmAP1 forms a protrusion on the L3 face at the proximal site that results in the formation of distinct grooves between the subunits (Figure S2; left panel), whereas Sso-SmAP2 displays a smoother surface due to the absence of the N-terminal extension (Figure S2; right panel). However, it remains to be shown whether this difference has a functional implication, e.g., with regard to the RNA binding properties of either protein. The analysis of the inter-subunit interactions revealed that the Sso-SmAP2 oligomeric assembly has a slightly larger interface area (874 Å^2^) than Sso-SmAP1 (834 Å^2^). Probability measures P_ΔG,IF_ of specific interfaces were derived from the gain in solvation energy upon complex formation, with P_ΔG,IF_ > 0.5 pointing to hydrophilic/unspecific and P_ΔG,IF_ < 0.5 to hydrophobic/specific interfaces. Sso-SmAP2 and Sso-SmAP1 present average P-values of P_ΔG,IF_ of 0.237 and 0.371, respectively, indicating an interaction-specific surface, which is more pronounced in the case of Sso-SmAP2.

The residues His40, Asn42, Arg66 and Asp68 located in loops 3 and 5 would form the uridine-binding pocket of Sso-SmAP1 (Figure 1D; blue) as inferred from the known structures of Af-SmAP1 and Pa-SmAP1 [18]. Interestingly, the corresponding region (Thr40, Asn42, Arg68 and Ser70) of Sso-SmAP2 displays modification(s). Thr40 replaces His40, which is responsible for stacking interactions with the uracil base (Figure 1D; magenta) [20]. In fact, the methyl group of Thr40 protrudes into the uridine-binding pocket, and thereby most likely interferes with uracil binding. The substitution of Asp68 by Ser70 might have a minor effect on RNA binding, given that only the main chain amide group participates in the interaction with RNA. Furthermore, the RNA binding site identified in the homologous Pa-SmAP1, which is located on the surface of the N-terminal α-helix [21], is not present in Sso-SmAP2. Tyr34 in Pa-SmAP1, which is responsible for stacking interactions with RNA, is replaced with Thr37 in Sso-SmAP2. Hence, Sso-SmAP2 shows two amino acid replacements, both of which are involved in RNA binding in other archaeal SmAPs.

**Figure 2:**
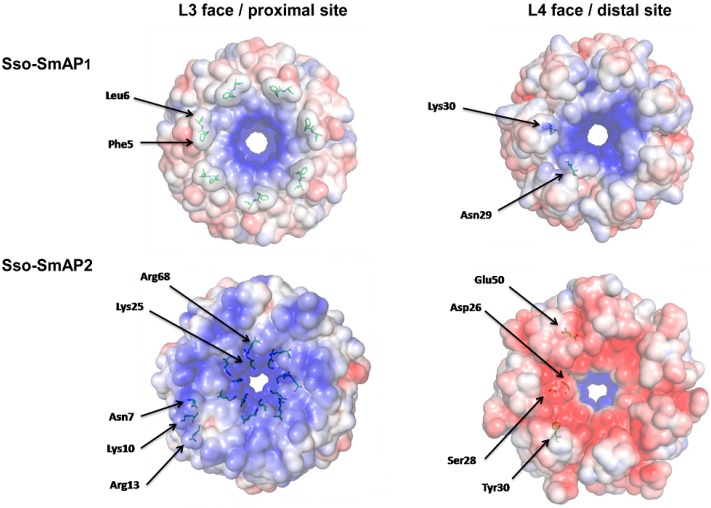
Electrostatic potential of the Sso-SmAP1 and Sso-SmAP2 surfaces. Poisson-Boltzmann electrostatic potential of the solvent accessible surface of Sso-SmAP1 and Sso-SmAP2. The calculations were done with the chains A to G of Sso-Sm2 (PDB code: 4XQ3) and chains A to G of Sso-SmAP1 (PDB code: 1TH7; [18], using the software APBS [34,35]. The scale ranges from −5 kT/e (red) to +5 kT/e (blue). The L3 face/proximal site (**left panel**) and the L4 face/distal site (**right panel**) of Sso-SmAP1 and Sso-SmAP2 are compared. The functional importance of the indicated amino acids is discussed in the text.

Next, we compared the electrostatic potential mapped on the solvent accessible areas of Sso-SmAP1 (Figure 2; upper panel) and Sso-SmAP2 (Figure 2; lower panel).The grooves created by the hydrophobic side chains of Phe5 and Leu6 at the N-terminus of Sso-SmAP1 create a charge separation on the L3 face/proximal site, which makes the periphery of the ring more negatively charged than the area in closer vicinity to the pore (Figure 2; left upper panel). In contrast, the L3 face/proximal site of Sso-SmAP2 is significantly more positively charged due to the presence of Asn7, Lys10 and Arg13 (Figure 2; left lower panel). Major differences of the electrostatic potential between both SmAPs were observed on the L4 face/distal site. Sso-SmAP2 is predominantly negatively charged (Figure 2, right lower panel), which is effected by residues Asp26, Ser28, Tyr30 and Glu50, whereas the presence of residues Asn29 and Lys30 confers a more positive charge distribution in Sso-SmAP1 (Figure 2, right upper panel). In both, Sso-SmAP1 and SsoAP2, the rim of the pore is positively charged on either site of the doughnut.

**Figure 3:**
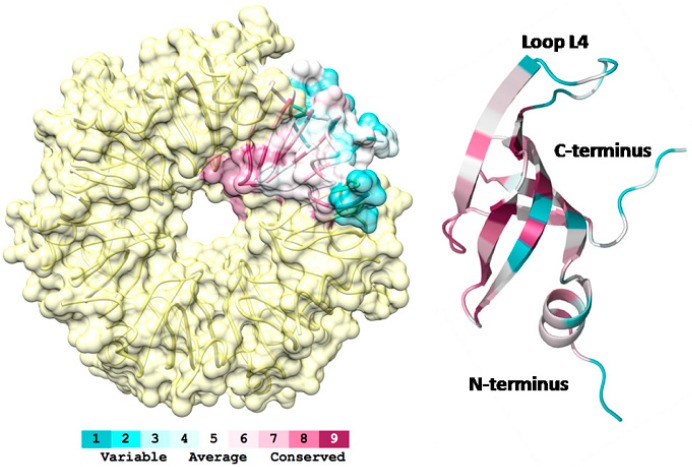
Evolutionary conservation of Sso-SmAP2. Ribbon diagram of a Sso-SmAP2 protomer colored in terms of evolutionary conservation of its amino acids (**right panel**). The conservation scores were calculated by Consurf [40] using the alignment of Sso-SmAP2 sequence against all archaea (Taxid: 2157) sequences [38,39]. The color code ranges from blue (non-conserved) to red (conserved). The N-terminal, C-terminal and Loop L4 regions stand out as non-conserved residues (blue). (**Left panel)**: Orientation of conserved patches within the heptameric ring. The most conserved residues are located in and in close vicinity of the central pore, whereas less conserved residues face the periphery of the heptameric ring.

To estimate the evolutionary conservation of amino acid positions in Sso-SmAP2, we aligned all known SmAP sequences from archaea using Blast and Cobalt [38,39], and mapped their conservation onto the Sso-SmAP2 structure, using the server Consurf [40]. As anticipated, this analysis revealed that the uridine-binding region on theL3face/proximal site is highly conserved (Figure 3). The degree of conservation progressively decreases from the interior (more conserved) to the outer rim of the heptameric ring (less conserved). Three regions are highly variable; the N-terminal region, loop L4 and the C-terminal tail (Figure 3), the latter of which displays the highest structural variability (Figure 1B). However, the function(s) of these regions is unknown for archaeal SmAPs. In the bacterial Sm-protein Hfq, the intrinsically disordered and flexible C-terminus is assumed to play a role in RNA interaction by tethering long and structurally diverse fragments [41,42]. The length of the N- and C-terminal domains is rather diverse in different archaeal SmAPs (FigureS3). For instance, the N-terminus of Sso-SmAP2 is six residues shorter than that of *Methanothermobacter thermautotrophicus*, belonging to the clade of e*uryarchaeaota*. However, the C-terminus of Sso-SmAP2 is five residues longer when compared to all other archael SmAPs with known 3D structure. It is thus conceivable that the length variation at the N- and/or C-terminus of SmAP proteins is related to their RNA binding mode.

### 3.2. Sso-SmAP1 and SsoAP2 Bind to Common and Distinct RNA Substrates

**Figure 4:**
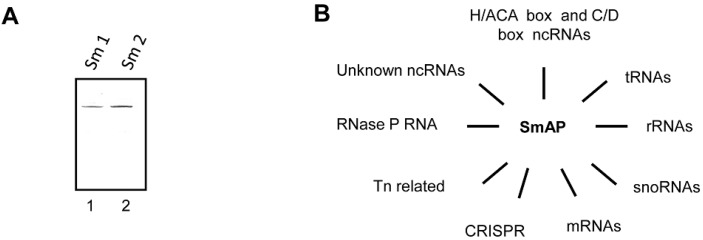
Purification of SmAPs from Sso and RNA classes found to be associated with them. (**A**) Purity of His-tagged SmAP1 (lane1) and SmAP2 (lane 2). SmAP1 and SmAP2 were purified from Sso strains PH1-16[pMJ05-SmAP1-His] and PH1-16[pMJ05-SmAP2-His] using His-affinity purification. One-tenth of the eluate was separated by SDS-PAGE followed by Coomassie staining of the proteins. The RNAs co-purifying with SmAP1 and SmAP2 were extracted with phenol/chloroform and precipitated. A cDNA library was generated with the extracted RNAs and analysed by RNASeq. The reads were aligned to the Sso P2 genome as described in Material and Methods. (**B**) Classes of RNAs co-purifying with SmAP1 and SmAP2. Among the co-purifying RNAs the following classes were discerned; (i) *trans*-encoded ncRNAs of unknown function [43], (ii) C/D box sRNAs, (iii) H/ACA box sRNA, (iv) CRISPR-, (v) Tn-related small RNAs, (vi) RNase P RNA, and (vii) L7Ae-associated RNAs as specified in [43,44,45]. In addition, a large number of mRNAs, tRNAs, snoRNAs and ribosomal RNAs were found to co-purify with both, Sso-SmAP1 and Sso-SmAP2.

The observations that (i) Sso-SmAP2 shows replacements of residues that are involved in RNA binding in other archaeal SmAPs (Figure 1), and that (ii) Sso-SmAP1 and SsoAP2vary in their surface electrostatic potentials (Figure 2) prompted us to test whether both Sso-SmAPs differ in terms of their RNA substrates. To address this both, Sso-SmAP1 and Sso-SmAP2, were isolated from Sso by using NiNTA magnetic beads. As judged by Coomassie staining of the eluates after electrophoretic separation, no other cellular proteins were co-purifying with either Sso-SmAP (Figure 4A). The identity of co-purifying RNAs was revealed by deep-sequencing (Table S1 and Table S2). Among the RNAs co-purifying with both, Sso-SmAP1 and Sso-SmAP2 (Figure 4B; Figure S3, Table S1_both), we identified *trans*-encoded unknown ncRNAs [43], C/D box containing sRNAs (which guide methylation sites in rRNAs or tRNAs), H/ACA box containing sRNAs, CRISPR- and Tn-related small RNAs, RNase P RNA and L7Ae-associated RNAs (Figure 4B) [43,44,45]. In addition, a large number of tRNAs, snoRNAs and ribosomal RNAs were identified that co-purified with both Sso-SmAPs (Figure 4B; Table S1). As SmAPs share a high homology with eukaryotic Sm proteins involved in splicing, and since in archaea introns occuronlyin tRNA and rRNA genes [46], these findings would be in line with the idea that the Sso-SmAPs are involved in rRNA/tRNA processing [2,17,47]. Similarly, the large number of tRNAs, rRNAs and tRNA- and rRNA-modifying RNAs like snoRNAs, C/D box-containing and H/ACA box-containing RNAs [44,48] co-purifying with both Sso-SmAPs point towards a role of the two Sso-SmAPs in processing/modifying these classes of RNAs. Moreover, the association of the Sso-SmAPs with RNase P RNA, a component of the tRNA-maturation endonuclease ribozyme RNase P [49], could hint towards a function in tRNA maturation, as previously inferred for Af-SmAP1 and Af-SmAP2 [19].

**Figure 5:**
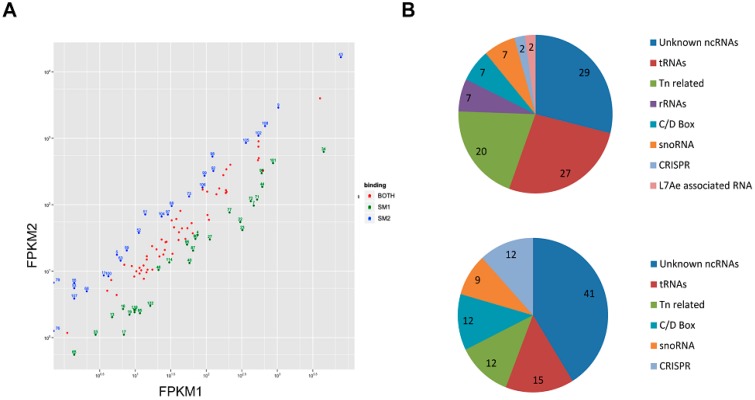
ncRNAs co-purifying with Sso-SmAPs. (**A**) Normalized read counts for ncRNAs binding to Sso-SmAP1 (FPKM1) and to Sso-SmAP2 (FPKM2). The reads from the alignment were counted for ncRNAs, and then normalized to obtain FPKM values (Fragment Per Kilobase of exon model per Million mapped reads). Many ncRNAs encoded in “*trans*” described in [43] were co-purifying with the Sso-SmAPs. NcRNAs binding to both, Sso-SmAP1 and Sso-SmAP2, are depicted with red dots. A threshold of a twofold difference in the FPKM values was used to select for ncRNAs that bind exclusively to either Sso-SmAP1 (green dots) or to Sso-SmAP2 (blue dots). (**B**) Functional classification of 28 ncRNAs enriched with Sso-SmAP1 (upper panel) and of 26 ncRNAs enriched with Sso-SmAP2 (lower panel).

In addition, we analyzed in more detail ncRNA candidates that were bound exclusively to either Sso-SmAP (Figure 5A). Only 2 transposon-related ncRNAs (ncRNA ID 76 and 79) [43] were found to be exclusively bound to Sso-SmAP2. For other associated ncRNAs a threshold of a twofold difference in the FPKM values (fc value) was used to select for ncRNAs predominantly binding to either Sso-SmAP1 (Table S1_Sm1 and Sm2; Figure 5A). Using this threshold we could identify Sso-SmAP1-specific ncRNAs (Figure 5A; green dots) and Sso-SmAP2-specific ncRNAs (Figure 5A; blue dots). The largest group of ncRNAs binding to Sso-SmAP1 is represented by ncRNAs of unknown function (29%) [43,44,45,50]. The second largest group is represented by tRNAs (27%), followed by snoRNAs, C/D box and rRNAs (7% each). Only 4% represented CRISPR- and L7Ae- associated RNAs (Figure 5B; upper panel). The largest group of ncRNAs binding to Sso-SmAP2 ncRNAs is again represented by ncRNAs of unknown function (41%). The second largest group is represented by tRNAs (15%), followed by transposon-related, C/D box-RNAs, CRISPR-RNAs (12% each) and snoRNAs (9%) (Figure 5B; lower panel).

The group of unknown ncRNAs associated with either SmAP1 or SmAP2 remains to be characterized. It will be interesting to study why they are specifically associated with one or the other SmAP, and if they target some of the mRNAs found to be associated with the respective Sso-SmAP (see below).

Transposon related ncRNAs are a dominant group among the Sso ncRNAs [43], which putatively regulate transposition [45]. One group of transposon-related RNAs represents classical anti-sense RNAs, which are complementary to transposon genes, and may serve to silence transposons [45]. Another group is represented by ncRNAs that exists in multiple copies, which appear to be remnants of transposition events that can act by anti-sense regulation of mRNAs as recently exemplified for an inorganic phosphate transporter gene [50].

**Figure 6:**
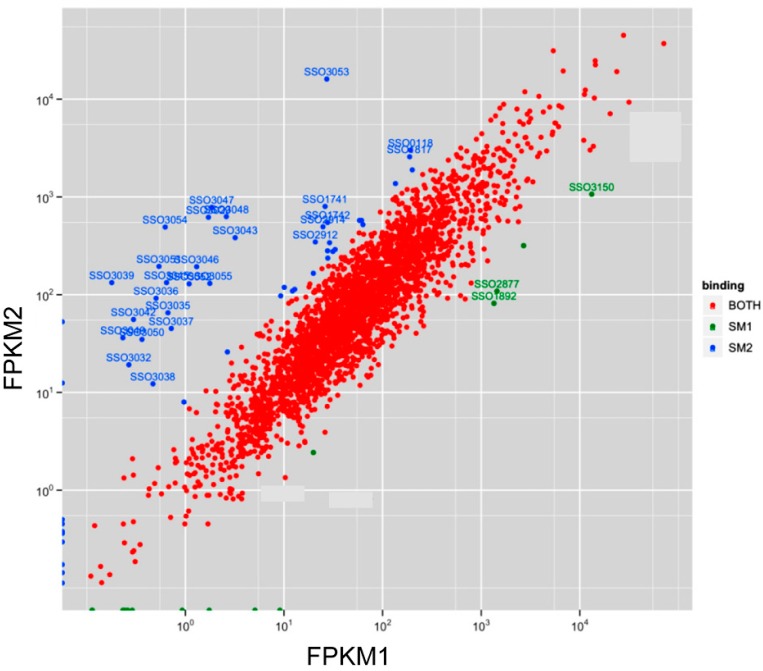
mRNAs co-purifying with Sso-SmAPs. Normalized read counts for mRNAs binding to Sso-SmAP1 (FPKM1) and to Sso-SmAP2 (FPKM2). Reads from the alignment were counted for the coding regions, and then normalized to obtain FPKM values (Fragment Per Kilobase of exon model per Million mapped reads). Red dots, mRNAs binding to both Sso-SmAPs. A threshold of an eightfold difference in the FPKM values was used to select for mRNAs that bind exclusively to either Sso-SmAP1 (green dots) or to Sso-SmAP2 (blue dots).

**Figure 7:**
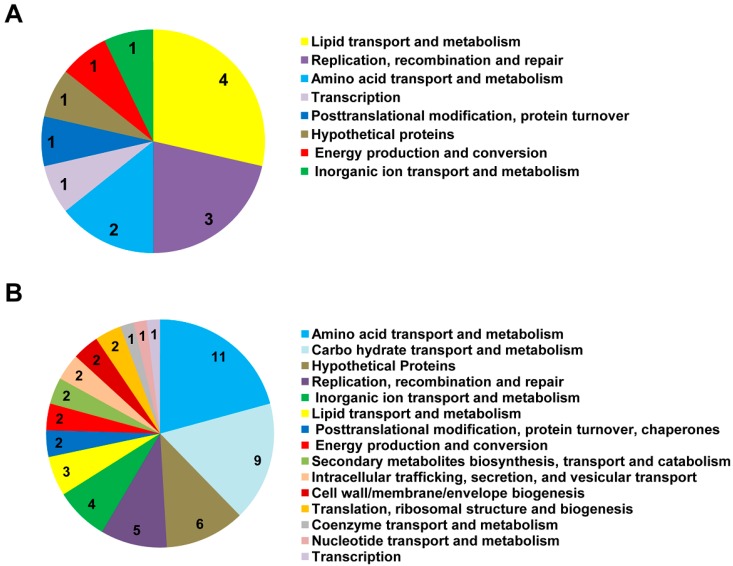
Functional categories of mRNAs binding to Sso-SmAPs. Functional classification of mRNAs bound to either Sso-SmAP1 (**A**) or to Sso-SmAP2 (**B**). A threshold of an eightfold difference in the FPKM values was used to select mRNAs binding to either Sso-SmAP1 or to Sso-SmAP2.This analysis revealed 14 and 53 mRNAs associated with Sso-SmAP1 and Sso-SmAP2, respectively. The color code depicts the encoded functions.

Surprisingly, most of the intron-containing tRNAs (http://gtrnadb.ucsc.edu/Sulf_solf/Sulf_solf-by-locus-txt.html) were more enriched in the Sso-SmAP1 preparation (Table S1; tRNAs marked in red). The binding of Hv-SmAP1 to tRNA has also been described for the clade of *euryarchaeota* [17]. Besides the ncRNAs, a large number of mRNAs associated with either Sso-SmAP (Table S2). The highest FPKM values for RNAs associated with both proteins were found for mRNAs encoding transporters and chaperones and proteins involved in energy metabolism and conversion (Table S3; highest FPKM). To identify specific Sso-SmAP1 and Sso-SmAP2-associated mRNAs, a threshold of an eightfold difference in the FPKM values (fc > 8) was used. Using this threshold, only 14 mRNAs were enriched in the Sso-SmAP1 preparation (Table S2_Sm1; Figure 6, green dots), whereas 53 mRNAs were found to be associated with Sso-SmAP2 (Supplementary Table 2_Sm2; Figure 6, blue dots).The 14 mRNAs bound to Sso-SmAP1 encode functions involved in lipid transport and metabolism (4%); replication, recombination and repair (3%); and amino acid transport and metabolism (2%) (Figure 7A). The majority of the 53 mRNAs bound to Sso-SmAP2 encode functions involved in different metabolic pathways, *viz* in amino acid transport and metabolism (11%) and in carbohydrate transport and metabolism (9%). 6% of the mRNAs encode (i) putative unknown proteins;(i) functions involved in replication, recombination and DNA repair (5%);(iii) factors involved in inorganic ion transport and metabolism (3%);and (iv) proteins associated with lipid transport and metabolism (3%) (Figure 6B).

## 4. Outlook

Up to today, the function of SmAPs remains largely unknown. In this study RNA substrates were identified for Sso-SmAP1 and Sso-SmAP2, which may provide hints as to their function in Sso. As intron-containing tRNAs were enriched with Sso-SmAP1, further studies may be directed towards a possible function of Sso-SmAP1 in tRNA/rRNA processing. In contrast, Sso-SmAP2 seems to bind predominantly to mRNAs and could therefore be involved in regulation of these in concert with the associated ncRNAs of unknown function. The most striking similarity is that all enriched mRNAs are involved in metabolic pathways. The comparison of the structure of both Sso-SmAPs revealed several differences with regard to the electrostatic surface potential and RNA binding sites, which might explain their association with distinct RNA substrates.

## Supplementary Materials

Supplementary materials can be accessed at: http://www.mdpi.com/2075-1729/5/2/1264/s1.
