# Supplementary material for: The Heptameric SmAP1 and SmAP2 Proteins of the Crenarchaeon Sulfolobus Solfataricus Bind to Common and Distinct RNA Targets
Source: Life (Basel). 2015 Apr 21;5(2):1264–81. doi: 10.3390/life5021264 (PMC4500138; doi:10.3390/life5021264)
Supplement: Supplementary file 1 [file life-05-01264-s001.zip › life-05-01264-supplementary.pdf]

Supplementary Materials

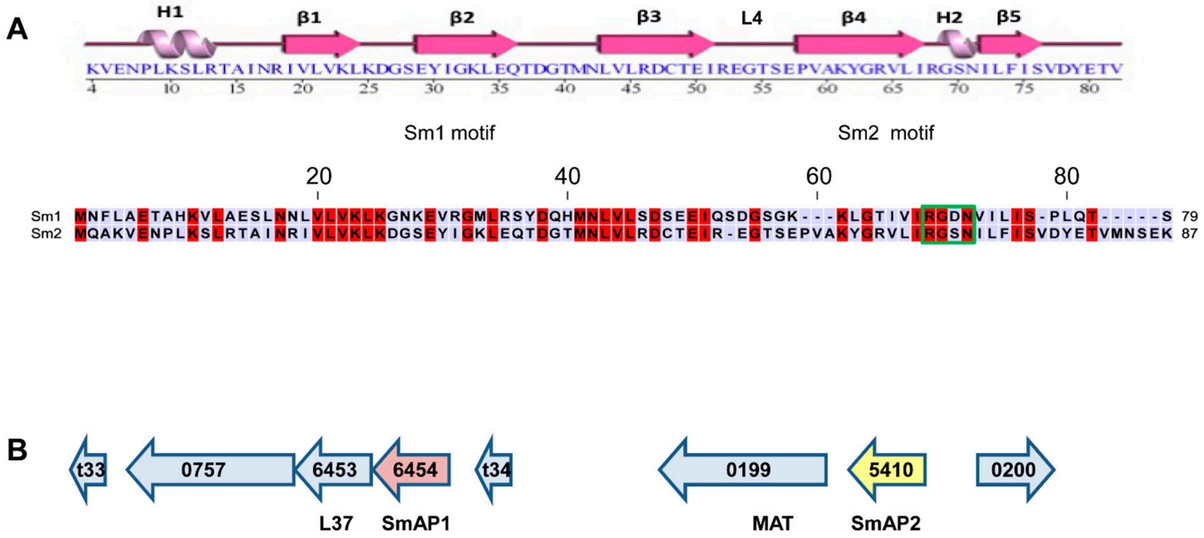

Figure S1. Secondary structure and conservation of SmAP1 and SmAP2. (**A**) Upper panel, the secondary structure of Sso-SmAP1 is shown superposed on the primary amino-acid sequence. β-strands and helices are represented by arrows and bars, respectively. The N-terminal a-helix is followed by five β-strands forming the continuous Sm-fold. The L4 loop links the Sm1 and Sm2 motif. Lower panel: conservation of Sso-SmAP1 and Sso-SmAP2. Like eukaryotic Sm proteins, both proteins contain a RGXX consensus sequence (green box), whereby X represents any charged amino acid. (**B**) Genomic organisation of the Sso-SmAP1 and Sso-SmAP2 gene loci. The Sso-SmAP1 gene (Sso 6545) is co-transcribed with ribosomal protein L37e gene and a spermidine synthetase gene. The Sso-SmAP1 operon is flanked by two tRNAs, t33 and t34, the latter of which contains an intron. The monocistronic Sso-SmAP2 (Sso 5410) transcript is transcribed in the same direction as a methionine adenosyl transferase (MAT) gene.

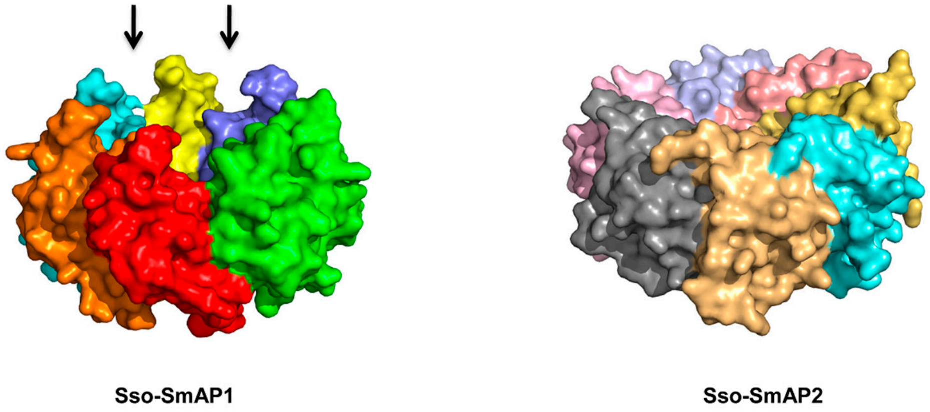

Figure S2. Representation of the Sso-SmAP1 and Sso-SmAP2 surfaces. The extended N-terminus of Sso-SmAP1 forms a protrusion on the L3 face/proximal site that results in distinct grooves (arrows) between the subunits (left panel) while Sso-SmAP2 displays a smoother surface (right panel). Each subunit is displayed in a different colour.

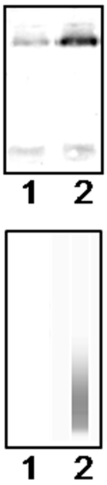

Figure S3. Western blot of SmAPs and co-purifying RNAs. (**Upper panel**): Western Blot from affinity-purified His-tagged SmAP1 (lane 1) and SmAP2 (lane 2) proteins using SmAP-protein specific antibodies. SmAP1 and SmAP2 were purified from Sso strains PH1-16[pMJ05-Sm1-His] and PH1-16[pMJ05-Sm2-His] using His-affinity purification. 1/10th of the eluate was separated by SDS-PAGE and transferred to a nitrocellulose membrane and detected with Sm-specific antibodies. With the antibody we could detect heptameric and some monomeric SmAPs. (**Lower panel**): The RNAs co-purifying with SmAP1 and SmAP2 were extracted with Phenol/Chloroform and precipitated. 1/10th of the extracted RNA was analysed with a Bioanalyzer before cDNA library preparation. Only in lane 2 the RNA was detectable.

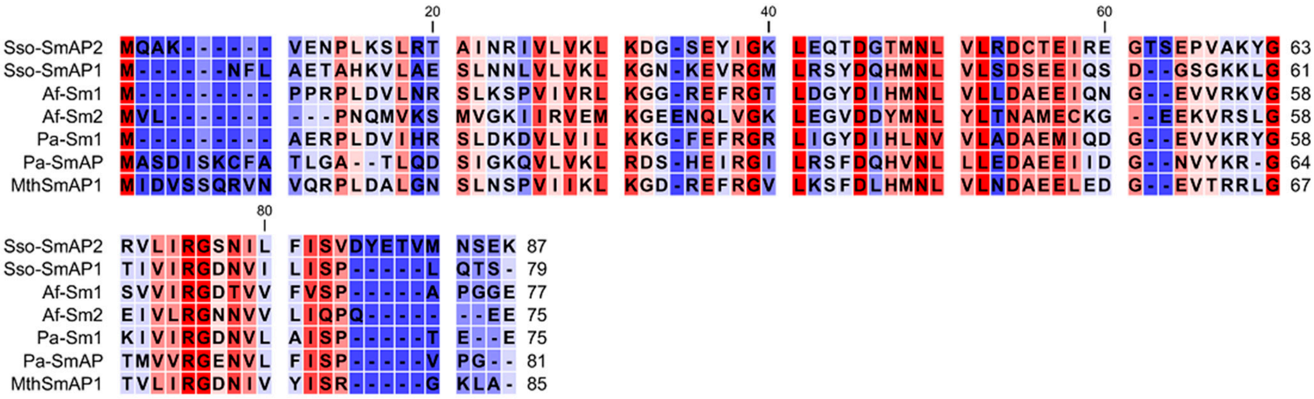

Figure S4. Alignment of different SmAPsThe protein alignment shows SmAPS from Sso-SmAP1 (Sulfolobus solfataricus—PDB code: 1TH7), Sso-SmAP2 (Sulfolobus solfataricus—PDB code: 4XQ3), Pa-SmAP—(Pyrobaculum aerophilum—PDB code: 1I8F), Pa-Sm1 (Pyrococcus abyssi—PDB code: 1M8V), MthSmAP1—(Methanobacterium thermautotrophicum—PDB code: 1JRI), Af-Sm2 (Archaeoglobus fulgidus—PDB code: 1LJO) and Af-Sm1 (Archaeoglobus fulgidus—PDB code: 1I4K). Conserved residues are depicted in red and non-conserved residues are shown in blue. For the alignment we used the CLC sequence viewer 6.6.2 software.
